# Early detection of health and welfare compromises through automated detection of behavioural changes in pigs

**DOI:** 10.1016/j.tvjl.2016.09.005

**Published:** 2016-11

**Authors:** Stephen G. Matthews, Amy L. Miller, James Clapp, Thomas Plötz, Ilias Kyriazakis

**Affiliations:** a Open Lab, School of Computing Science, Newcastle University, Newcastle upon Tyne, NE1 7RU, UK; b School of Agriculture, Food and Rural Development, Newcastle University, Newcastle upon Tyne, NE1 7RU, UK

**Keywords:** Animal behaviour, Automated detection, Health and welfare, Pigs, Sensors

## Abstract

Early detection of health and welfare compromises in commercial piggeries is essential for timely intervention to enhance treatment success, reduce impact on welfare, and promote sustainable pig production. Behavioural changes that precede or accompany subclinical and clinical signs may have diagnostic value. Often referred to as sickness behaviour, this encompasses changes in feeding, drinking, and elimination behaviours, social behaviours, and locomotion and posture. Such subtle changes in behaviour are not easy to quantify and require lengthy observation input by staff, which is impractical on a commercial scale. Automated early-warning systems may provide an alternative by objectively measuring behaviour with sensors to automatically monitor and detect behavioural changes. This paper aims to: (1) review the quantifiable changes in behaviours with potential diagnostic value; (2) subsequently identify available sensors for measuring behaviours; and (3) describe the progress towards automating monitoring and detection, which may allow such behavioural changes to be captured, measured, and interpreted and thus lead to automation in commercial, housed piggeries. Multiple sensor modalities are available for automatic measurement and monitoring of behaviour, which require humans to actively identify behavioural changes. This has been demonstrated for the detection of small deviations in diurnal drinking, deviations in feeding behaviour, monitoring coughs and vocalisation, and monitoring thermal comfort, but not social behaviour. However, current progress is in the early stages of developing fully automated detection systems that do not require humans to identify behavioural changes; e.g., through automated alerts sent to mobile phones. Challenges for achieving automation are multifaceted and trade-offs are considered between health, welfare, and costs, between analysis of individuals and groups, and between generic and compromise-specific behaviours.

## Highlights

• Recent developments in automatically detecting compromised pig health and welfare.
• Five categories of behaviour are reviewed.
• Behaviours mapped to sensors that are feasible for automated detection.
• Progress towards levels of automation through detection, monitoring and fully automatic detection of behavioural change.
• Challenges for automated detection of behavioural changes are multifaceted and require trade-offs to develop such systems.

## Introduction

In recent years, there has been increased concern over pig welfare under intensive farming systems, with the scientific consensus being that an animal's welfare state should be enhanced (Mellor, 2016). Health and welfare compromises in pigs have wide-ranging consequences, including system profitability and sustainability. Early detection of health and welfare compromises will increase treatment success, may contain problems, and enhance pig welfare and system sustainability. However, early detection typically requires human observation, which can be subjective, and examination of individuals to detect salient changes and clinical signs (Radostits et al., 2007a). Furthermore, subclinical illness, by definition, is invisible and usually only detected at slaughter, which creates a significant challenge for early detection under commercial conditions.

One way to achieve early detection of health and welfare compromises in animals is to utilise behavioural changes. Such changes precede clinical signs of disease or injury and affect animal performance (Hulsen, Scheepens, 2006, González et al, 2008, Kyriazakis, Tolkamp, 2010). Quantification of behaviour and its changes by staff can be time consuming, subjective, and impractical, particularly on large scale farms (Hemsworth et al., 2000). Automation is defined as operating or acting, or self-regulating, independently, without human intervention (Nof, 2009), which is a recent trend to overcome this (Cornou and Kristensen, 2013). Automation in commercial piggeries presents substantial challenges (Banhazi et al., 2015) for sensor and computer hardware, sensor data processing, computer vision, and machine learning.

The aims of this paper are: (1) to identify behaviours with associated quantifiable effects that have diagnostic value for automatic detection of health and welfare compromises; (2) to review sensors capable of measuring behaviour; and (3) review the current status of automation that may allow such behavioural changes to be captured, measured, and interpreted. Previous reviews have identified automated sensing approaches to quantify physiology (Eigenberg et al., 2008) and assess automated sensing of behaviour specifically for early warning of tail biting (Larsen et al., 2016). We expand the scope to any health and welfare compromise that has diagnostic value and focus on rearing pigs in housed environments.

## Pig behaviours associated with compromised health and welfare

Changes in the behaviour of commercial pigs can be the result of various challenges including the inability to express normal behaviour as dictated by the farm environment, disease, or injury. Such changes are in direct conflict with the Five Freedoms,^1^ and do not adhere to the new five domains model (Mellor, 2016), and must be addressed in order to improve the welfare of the animals and to enhance system sustainability. Behavioural changes during health and welfare challenges can have an evolutionary basis (Hart, 1990, Kyriazakis et al, 1998), or are the inevitable consequence of the challenge. For example, exposure to pathogens is associated with changes in feeding behaviour that may be of benefit (Kyriazakis et al., 1998), whereas crowding may be viewed as a risk factor for tail biting (Edwards, 2006). Farmed livestock, including pigs, are unable to exhibit all behaviours that would normally be expected to reflect the consequences of compromised health and welfare (Hart, 1990), due to the way they are husbanded. However, certain behaviours are still evident, although they may no longer serve their original function (Kyriazakis and Tolkamp, 2010). Below, we provide an overview of pig behaviours reported to change as a result of compromised health and welfare. Five behavioural categories that may inform the development of automated detection systems are shown in Table 1.

## Behavioural categories

### Daily activity budgets

Activity budgets relate to the use of an animal's time and are often associated with fulfilling specific requirements for survival and growth for pigs (Maselyne et al., 2014a) and other species (Uzal and Ugurlu, 2010). Activities include moving, standing or lying, feeding, drinking, social and aggressive behaviours (Maselyne et al., 2014b). Growing pigs show a diurnal rhythm of activity (Costa et al, 2009, Chung et al, 2014), and typically display increased activity from social and exploratory behaviour follows feeding in growing pigs, with approximately 70% of their time inactive (Maselyne et al., 2014a).

Changes in activity can be reported as frequency, duration, time of day, sequences of behaviours, and complexity of those sequences. Significant differences in activity budgets were found in compromised pigs, such as after infection (Escobar et al, 2007, Reiner et al, 2009), prior to outbreaks of tail biting (Statham et al., 2009), and after stress induction (Salak-Johnson et al., 2004). Enriched environments reduced the time that pigs sat compared to barren environments (Studnitz et al., 2007). The method of enrichment provision can alter the amount of the activity budget spent interacting with it. The rotation between various enrichments or provision of two types of enrichment concurrently, can increase the amount of the daily activity budget spent, although ultimately habituation does still occur (Trickett et al., 2009). Low behavioural complexity (increased regularity and decreased randomness) was observed in stressed pigs in the form of more structured sequences between standing or walking and other postures (Rutherford et al., 2006).

### Feeding, drinking, and elimination behaviours

Feeding behaviour in pigs is influenced by enrichment (Jensen et al, 1993, Zebunke et al, 2013), management practices (Figueroa et al., 2013), and infection (Escobar et al, 2007, Reiner et al, 2009). A voluntary reduction in daily food intake (Kyriazakis, 2014) is common to many infections and often an early sign of disease in farm animals (Munsterhjelm et al., 2015). The degree of reduction can be influenced by pathogen type (Kyriazakis and Houdijk, 2007) and relate to the challenge's subclinical or clinical nature (Kyriazakis, Houdijk, 2007, Sandberg et al, 2007). In other cases, changes in feeding and drinking behaviours may occur without reductions in daily food or water intake (González et al., 2008), but instead relate to changes in frequency and duration of eating and drinking (Tolkamp et al., 2011). Drinking increases in some metabolic diseases and also in response to dehydration resulting from diarrhoea (Madsen, Kristensen, 2005, Seddon, 2011), stress (Averos, 2007), and high ambient temperature (Rushen et al., 2012). Increased stocking density was shown to cause pigs to drink more at each opportunity, but less frequently (Andersen et al., 2014). The frequency and duration of elimination, drinking, and lying behaviour was shown to predict early stages of bacterial infections (Krsnik et al., 1999). *Salmonella* infection in pigs results in reduced feeding and drinking behaviours (throughout the 4 week post-infection period) and a decrease in the time spent standing and sitting (by week 4 post-infection) compared to controls (Ahmed et al., 2015). Changes in urinary frequency or volume may also be used as indicators of impaired health (Radostits et al., 2007b). Additionally, pigs become less clean in their elimination behaviour above a critical environmental temperature (Aarnink et al., 2006).

### Behaviours associated with posture and locomotion

Posture and locomotion can be influenced by skeletal and visceral diseases (Radostits et al., 2007c). Lameness is a major cause for culling breeding pigs (Tarrés et al., 2006), and may be caused by infection, degeneration, or trauma to one or more limbs. Scoring lameness severity (D'Eath, 2012) can be useful for monitoring treatment outcomes; e.g., in dairy cattle (Chapinal et al., 2010), and measuring lameness incidence on farm. Health compromises may also result in postural changes; e.g., the sawhorse stance, guarding and dog sitting can be shown with abdominal pain (Radostits et al., 2007d). *Salmonella* infections caused pigs to spend more time lying sternally, standing, and sitting than in control pigs (Rostagno et al., 2011). Tail carriage is also a useful barometer of health, with tails curled upwards indicating active pigs (Kleinbeck and McGlone, 1993), and tails pressed between hind legs are linked with stress or pain (Kiley-Worthington, 1976, Noonan et al, 1994). Blindness and brain disorders can be linked with locomotion, such as circling or walking into objects (Radostits et al., 2007e).

### Social behaviours

Changes in social behaviour can provide indicators of health and welfare, such as when individuals become isolated (Reimert et al., 2013). Cohesive or clustering behaviour may also be displayed to maintain thermal comfort from pyrexia (Cook et al., 2015) or environmental conditions (Costa et al., 2014). Low ambient temperature causes pigs to huddle for mutual heat and reduce heat loss (National Research Council, 1981), while high ambient temperature influences lying behaviour with pigs spreading out (Jackson and Cockcroft, 2007a).

Vocalisations in pigs have been studied as an indicator of welfare. Low-pitched vocalisations, e.g., grunts, maintain social contact with conspecifics. Deviations from these usual vocalisations could be used in welfare assessment. Social isolation, castration, and weaning were all procedures that resulted in high call rates, with high frequency, duration, and amplitude (Manteuffel et al., 2004). Additionally, feed deprivation can be detected by analysing the number of screams (Vandermeulen et al., 2015), and the stressful procedure of handling piglets also results in changes in vocalisations (Moura et al., 2008).

Tail biting is a significant problem in pigs that has both welfare and economic consequences. Risk factors associated with tail biting have been identified as gender, herd size, density, age and weight, floor, feed, state of health, enrichment, air quality, and genetics (Schrøder-Petersen, Simonsen, 2001, Taylor et al, 2010, Sonoda et al, 2013). Behaviours exhibited include: ‘two-stage’ tail biting, gentle tail manipulation of another pig's tail followed by dental manipulation; ‘sudden-forceful’ tail biting, grabbing and yanking tails; and ‘obsessive’ tail biting, repeatedly grabbing and yanking tails (Taylor et al., 2010). Following a wound to the tail, the presence of blood stimulates further tail biting, indicating how an initial minor tail injury can ultimately result in an unpredictable and large increase in biting behaviour (Schrøder-Petersen and Simonsen, 2001).

Specific changes in the behaviour of pigs, prior to a tail biting outbreak may be used to predict cases and allow timely intervention. General activity levels are significantly higher 4 days prior to a tail biting outbreak, with more pigs standing and fewer pigs sitting or lying inactively than in matched control groups (Statham et al., 2009). Increased levels of chewing activity post-weaning at 8–11 weeks correlate with an increase in likelihood of tail biting in weeks 8–11 and again in weeks 16–21 (Ursinus et al., 2014). Tail biting outbreaks were less likely to occur when significantly fewer pigs held their tails in a tucked under position when at 11 weeks (Statham et al., 2009). Low feeding frequencies at the group level, up to 9 weeks prior to the first injury have also been identified (Wallenbeck and Keeling, 2013). Additionally, feed intake has been shown to decrease 20 days prior to being tail bitten (Munsterhjelm et al., 2015).

### Disease-specific behaviours

Various non-specific behavioural changes may be seen across a range of conditions in commercial pigs; e.g., a reduction in food intake (Jackson and Cockcroft, 2007b) and typical sickness behaviour (Dantzer, 2004). However, a result of some compromises to health, also lead to disease-specific changes that can be used for diagnosis and determining appropriate intervention. Examples include lameness in foot rot (Jackson and Cockcroft, 2007c), coughing during respiratory infections (Ferrari et al., 2008), high-pitched squealing in oedema disease (Jackson and Cockcroft, 2007d), and scratching in pruritic mange (Taylor, 1999).

### Towards quantification of behavioural changes

The behaviour of pigs is a valuable indicator of farm health and welfare status, reflecting animal responses to these changes and the surrounding environment. Measuring individual and group behaviours has merit in specific contexts. Behaviour of individuals can identify posture, locomotion and behaviour complexity, and can be particularly suitable when response to health and welfare compromise can be based on phenotype. Observing individuals is labour intensive and may be impractical for monitoring in a commercial environment. Whilst groups exhibit social behaviours, such as thermal comfort and tail biting, individual behaviour is also relevant for social behaviour, such as stress reactivity. Group approaches typically create a group-level of normal behaviour, such as for feeding, drinking and elimination. As previously indicated, subtle changes in behaviour, such as the frequency of standing episodes (Statham et al, 2009, Rostagno et al, 2011, Ahmed et al, 2015) may also be associated with health and welfare compromises. However, these changes are detected when the magnitude significantly changes when it may be too late from a diagnostic perspective. These issues may be overcome with the automated recording of behavioural changes. Detecting and analysing often subtle behavioural changes as health and welfare indicators, go beyond the classical paradigm of disease detection through clinical signs.

## Sensors for measuring pig behaviour

In order to successfully automate detection of compromised health and welfare, the appropriate choice of technology with correct application to measuring animal behaviour is crucial. Sensors that are commercially available and technically feasible for measuring behaviour are reviewed according to sensor modalities.

### Audio

Microphones convert changes of sound pressure into electrical signals, which are then captured by specific audio equipment (sound cards with multi-channel analogue-to-digital converters and amplification hardware) and processed as digital signals in standard computers. Automated processing techniques typically aim at detecting and classifying specific acoustic events, such as coughing, sneezing, screaming, and barking, and optionally, these can be enhanced by automated localisation of sound sources. The actual assessment of audio data is then usually based on spectral analysis; i.e., the automated decomposition of acoustic signals into (bands of) relevant frequencies and subsequent processing. Detecting (Chedad et al., 2001) and classifying sick pig coughs is possible, even if there is a small difference in frequency between screams and sneezes (Exadaktylos et al., 2008). Detecting different types of coughs associated with pig wasting diseases has also been demonstrated (Chung et al., 2013). The STREMODO system automatically measures the duration and intensity of stress from vocalisations (Manteuffel, Schön, 2002, Schön et al, 2004).

For automated sound source localisation either specialised, directed microphones, or groups of microphones, so-called microphone arrays, are used (Hennecke et al., 2009). Such systems are then used to contextualise animal behaviours to acoustic events outside the pen (e.g., loud tractor noise; Brouček, 2014), or to locate these (e.g., coughing animals; Silva et al., 2008). Audio also has the potential to measure the farming environment, which can provide indications for external stimuli that may have behavioural impacts (Marx et al, 2003, Brouček, 2014).

### Visual

Video is widely used in agriculture (Davies, 2009) where automated data processing techniques typically aim at recognising objects, understanding scenes, and tracking motion of objects and cameras (Szeliski, 2011). Light passes through a camera lens and typically onto a sensor that has an array of cells to convert light (photons) into electrical charge (electrons). The result is a digital image formed from a grid structure of pixels produced from the sensor's cells.

Measures of general pig activity have been inferred from differences in pixels between consecutive images (Kashiha et al., 2013c) and fraction of movement on floor space (Leroy et al., 2006). Pixel differences between consecutive images are capable of detecting fast pig movement that may indicate aggressive behaviour (Viazzi et al., 2014). However, these movements are not always attributed to aggressive behaviour and may indicate other behaviours such as playing and chasing (Viazzi et al., 2014). Leveraging a similar approach based on pixel differences, it is possible to extract detail about aggression, such as low, medium, and high level (Oczak et al., 2014). A method to automatically detect head-to-head (or body) knocking and chasing has been demonstrated with a 3D camera (Lee et al., 2016).

Measuring behaviour based on pixel differences between consecutive images provides a measure of the group rather than individuals (Rushen et al., 2012). Detecting individual pigs, with methods such as segmentation (separating pigs from other pixels; McFarlane and Schofield, 1995) and ellipse fitting (Kashiha et al., 2013b), enables pig location to be tracked. Tracking location of an unusually active pig (through apomorphine treatment) showed the total distance travelled to be higher (Lind et al., 2005). Tracking multiple pigs in real time has been prototyped and demonstrated in a simulation and actual pig pen to track locations of three pigs for 8 minutes without losing identities (Ahrendt et al., 2011). Segmenting the proportion of pigs in regions of interest in a pen was validated with manual observation (Nilsson et al., 2015), while measuring the spatial distribution of pigs in colour video provides an indication of thermal comfort (Shao and Xin, 2008).

Colour video has been used to measure locomotion in 2D by overlaying multiple images of motion to assess structure and patterns of movement (Kongsro, 2013) and a Vicon^2^ motion capture system uses multiple cameras to track reflective markers placed on pigs to measure locomotion in 3D (Stavrakakis et al., 2014). More practical solutions for a commercial environment use a single camera to measure lameness in 3D (Stavrakakis et al., 2015) and the height of pigs (i.e., lying down; Kulikov et al., 2014).

Non-invasive measurement of surface temperature of pigs from infrared video (McManus et al., 2016) has supported the evaluation of automated assessment of thermal comfort measured from the spatial distribution of pigs (Cook et al., 2015). Measuring temperature non-invasively, such as with infrared cameras, can improve welfare by reducing stress from restraining animals (Soerensen and Pedersen, 2015). Benefits of infrared are the large contrast in infrared intensity between pigs and the background environment can aid detection of pigs, especially at night (Costa et al., 2014).

Vision data may require considerable processing and there have been studies into the trade-off between accuracy of activity (motion detection) and computational processing requirements (Chung et al., 2014). Software challenges include detecting pig locations by separating touching or adjacent pigs and choosing appropriate features to recognise individual pigs (Sa et al., 2015). In addition, cameras are susceptible to the typically hostile environment of pig units with dust and damage from ammonia (Ahrendt et al., 2011), although this can potentially be negated through ingress protection enclosures and maintenance.

### Other modalities

Water flow sensors can infer group drinking behaviour (Madsen and Kristensen, 2005) and are reported to be more accurate than experienced observers (Meiszberg et al., 2009). However, there are challenges such as installing sensors in existing plumbing, variable water flow rates, short drinking bouts (Maselyne et al., 2015a), assumed drinking behaviour when a snout is in an outlet (Kashiha et al., 2013a), and water wastage without apparatus to collect wasted water (Andersen et al., 2014).

Radio-frequency identification (RFID) at feeding and drinking areas has been used to measure occurrence and duration of individual pigs' feeding and drinking behaviour (Fernández et al, 2011, Andersen et al, 2014, Maselyne et al, 2015a, Maselyne et al, 2015b). An RFID system requires an RFID transponder (ear tag) and an RFID antenna or receiver (located at the feeder or drinker). Low-frequency RFID cannot identify individuals when multiple transponders (on pigs) are close to one receiver. This is overcome by ensuring only one pig is present (Brown-Brandl and Eigenberg, 2011); e.g., at Skiold Acemo^3^ electronic feeding stations, or combining a multiplexer with high-frequency RFID to distinguish individual pigs in a group (Maselyne et al., 2014b). Combining RFID and water flow sensors has greater accuracy than water flow sensors alone (Maselyne et al., 2015a), but it depends on RFID position and orientation (Maselyne et al., 2014c). Exploiting RFID for measuring location-based behaviours, such as daily activity budgets, is restricted by the short communication range between transponder and antennae or receiver due to the limited power source of RFID.

Interaction with enrichment ropes has also been quantified by measuring chewing of a sealed air chamber attached to the rope (Feddes et al., 1993) and measuring when the rope moves (conductive metal pin on rope makes contact with metal loop around rope to complete path of electrical circuit; Zonderland et al., 2003).

Accelerometers attached to pig ears combined with body temperature sensors automatically detected an infection 1–3 days prior to sampling techniques (Martínez-Avilés et al., 2015).

Pressure mats have been shown to provide an objective method to measure gait, which has potential for early detection of lameness (Meijer et al., 2014).

## Monitoring behaviour and automatically detecting change

The ability to detect behaviour provides the basis for monitoring behaviour and automatically detecting behavioural changes. Full automation means operating with complete human independence and in an unsupervised way. For example, a temperature control system measures the current temperature and automatically changes the temperature to maintain a target temperature. We draw the distinction between monitoring a system (by staff) and full automation based on automated detection of behavioural change. The key difference is that monitoring requires staff to actively identify changes and make management decisions based on these, whereas automated detection (full automation) has the ability to send alerts to staff advising them of behavioural changes and potentially identification of the compromise and rectification. Previous reviews have focused on physiology (Eigenberg et al., 2008) and the ability to measure behaviour (Frost et al, 1997, Wathes et al, 2008, Cornou, Kristensen, 2013); however, this review focuses on digital automation specifically for monitoring behaviour and detecting behavioural change. Table 2 shows the current levels of automation for each behavioural category.

### Daily activity budgets

Circadian rhythm of group pig activity was identified and monitored over multiple days (Chung et al., 2014), where pig activity was measured from motion detection of difference in pixels between consecutive images. Diurnal group drinking behaviour was measured with water flow sensors specifically to monitor deviations leading to diarrhoea outbreaks (Madsen and Kristensen, 2005) and individuals were identified by combining with RFID (Andersen et al., 2014). Neither measures all activities to model a daily activity budget but these approaches do measure one or more behaviours over time for automated monitoring.

### Feeding, drinking, and elimination behaviours

Commercial feeding stations provide monitoring of individual pig feeding behaviour by using RFID, such as the individual feed intake recording of pigs in group housing (IVOG) system (Bruininx et al., 2001; Hokofarm Group B.V.^4^ formerly Insentec B.V.^5^), the Feed Intake Recording Equipment (FIRE),^6^ and Eliskool 2/Elistar 2/Tristar systems.^7^ Commercial systems for monitoring group water consumption are available as modules in the I-BOX 360° management system^8^ and the Farmex remote monitoring system.^9^ Monitoring water consumption of groups of pigs with water flow demonstrated automated analysis that was able to detect behavioural changes in diurnal drinking patterns 1 day before physical signs of diarrhoea were observed (Madsen and Kristensen, 2005). Similarly, automated detection of changes in feeding behaviour at a feeding station was able to predict tail biting as early as 9 weeks before its onset (Wallenbeck and Keeling, 2013).

Other automation systems have the ability to alert staff to system faults and threshold conditions being met, such as Farmex. Multiple sensor modalities (camera and RFID) can also be used to provide alerts to the degraded system performance when one sensor functions abnormally (Gregersen et al., 2013).

Assessing changes in frequency of entering an elimination zone was reported as a computer system to demonstrate the potential for identifying different behaviours exhibited by sick pigs (Zhu et al., 2009).

### Behaviours associated with posture and locomotion

The number of pigs in a cluster or huddle of pigs provides a measure of spatial distribution that demonstrated increased clustering or huddling in vaccinated pigs (Cook et al., 2015), and changes in pig lying behaviours under different environmental temperatures (Nasirahmadi et al., 2015).

Detecting changes in gait and stride kinematic from a 3D camera is possible (Stavrakakis et al., 2014); however, there are practical challenges, such as guiding pigs along a walk way (Stavrakakis et al., 2014), staff requirement, and sufficient unoccupied area in a commercial environment (Kongsro, 2013), which may be limited by stocking density.

The eYeNamic system^10^ (Leroy et al., 2006) monitors group activity in zones of a pen by quantifying pixel differences in consecutive images. eYeNamic measures general activity from movement and can be interpreted for assessing aggression (Costa et al., 2007), responses to dust concentration (Costa et al., 2009), enrichments (Ismayilova et al., 2013), and climatic variation (Costa et al., 2014). A similar approach using commercially available, general-purpose motion detection software measured movement in hot spots of a pen to automatically detect behavioural changes (Martínez-Avilés et al., 2015).

### Social behaviours

The concept of automatic analysis of aggressive behaviours to identify behavioural changes has been suggested as an early warning indicator (Oczak et al., 2012). This allows actuators (undefined but based on sound or smell) to influence changes in pig behaviour and reduce aggression levels (Oczak et al., 2012). This is not yet reported to be developed and operational.

### Disease-specific behaviours

The Pig Cough Monitor is a system that monitors pig coughs (Vandermeulen et al, 2013, Hemeryck, Berckmans, 2015, Hemeryck et al, 2015), and is now a commercial product called Respiratory Disease Monitor.^11^ Case studies reported increases in coughs when piglets were moved, which was attributed to stress, instances of changes in building temperature, and also problems with ventilation systems. The cough index is a group-level measure based on the number of coughs across a group of pigs in a day. A similar approach refined the cough index by considering the number of pigs (Nathues et al., 2012). It was recommended to combine the cough index with seroprevalence measurements and veterinary skills for diagnosis, which may suggest limited usability for automation (Nathues et al., 2012).

## The way forward: trade-offs in automated detection

Current progress towards automated detection of health and welfare compromises indicates that three categories of approaches to automation are emerging (see Table 2). The first category reports only on detecting behaviours using sensors. This can be straightforward for measuring some behaviours, such as a single instance of feeding (with RFID), but more challenging for measuring other behaviours such as aggression (with video). The next category applies the detection method over time, records behavioural data, and presents these to staff for monitoring of potential problems, typically in graph form (e.g., on a mobile phone). This category enables identification of behavioural changes, but requires farm staff to identify the change, such as decreased feeding between days and between pens. The final category automatically analyses the recorded behaviour over time to detect behavioural changes, and automatically sends alerts to staff advising them of behavioural changes and potentially identification of the compromise and rectification. The key difference is that the system identifies the behavioural change and not the farm staff.

Most progress in automation has focused on the second category (monitoring behaviour), and there are few reports on the third category (automatically detecting behavioural changes). In three cases, the data analysis methods were capable of automatically detecting behavioural changes in drinking behaviour from water flow sensors before diarrhoea (Madsen and Kristensen, 2005), in feeding visits and consumption with RFID feeding stations before tail biting (Wallenbeck and Keeling, 2013), and movement activity from video before clinical signs of swine fever (Martínez-Avilés et al., 2015). Monitoring behaviour measures and reports behaviour, but these three cases use more advanced approaches of applying data analysis to automatically detect behavioural changes. Each behavioural category (see Table 2) has an approach for monitoring behaviour with the exception of social behaviours, which could present more technical challenges for some sensors such as cameras.

Challenges for automated detection of behavioural changes are multifaceted and require trade-offs in developing such systems. The ultimate trade-off is between a system that maintains *health and welfare* of every pig and the *costs* to achieve this, such as the initial and maintenance costs of technology, and the value provided by this information (Cornou and Kristensen, 2013). As with any diagnostic tool, system acceptance depends on specificity and sensitivity. Similarly, reduced observations from staff and system reliability and robustness in a farm environment (Banhazi et al., 2015) raise ethical concerns and must not compromise animal health and welfare.

Trade-offs between *general* and *specific* health and welfare compromises impact the ability to monitor many compromises and the associated cost. Measuring generic behaviour can lead to monitoring and detection of multiple behavioural changes (e.g., eYeNamic measured general activity of a group and was utilised in multiple behaviour studies Costa et al, 2007, Costa et al, 2009, Costa et al, 2014, Ismayilova et al, 2013). Measuring specific behaviours with individual sensors may provide less value from the cost of technology; however, the choice of sensor may facilitate measuring multiple behaviours, such as the potential for video cameras to measure all of the behavioural categories in Table 1 with the exception of the disease-specific category.

Trade-offs between analysis of *individuals* and *groups* impact the ability to monitor each pig and the associated cost. Some sensors have the advantage of identifying pigs, such as RFID in feeding stations, while sensors that do not continuously identify pigs provide other advantages, such as location in pen and locomotion from video cameras. Analysing individuals may require a sensor per animal, such as RFID transponders; however, analysing the group may require just one sensor (e.g., the Pig Cough Monitor). The ultimate choice will depend on the aim; e.g., measuring groups may be more suitable for hundreds or thousands of pigs, but, for pedigree pigs, the emphasis might be on the individual and the consequent cost may be justified.

## Conclusions

Automation is a new tool within agriculture that has the potential for detecting behavioural changes as a result of health and welfare compromises. Automation technologies have the potential to enable and advance scientific knowledge. This review identified important cases of available automated technologies that allow detection of small deviations in diurnal drinking, deviations in feeding behaviour, monitoring coughs and vocalisation, and monitoring thermal comfort. Furthermore, the review identified five behavioural categories, which precede subclinical and clinical signs, and one or more sensors for objectively measuring behaviour in each category. Approaches to monitoring these behaviours have been reported in the scientific literature except for social behaviours, which may be addressed by new sensing approaches, new sensor modalities, and more advanced data processing methods. Most approaches monitor behaviour and require a person to detect behavioural changes, so a system with a higher level of autonomy that automatically raises alerts can support welfare, profitability, and sustainability. The challenges for automation are multifaceted and consideration of the trade-offs is recommended for developing automated approaches and can also support successful uptake in commercial piggeries.

## Conflict of interest statement

None of the authors of this paper has a financial or personal relationship with people or organisations that could inappropriately influence or bias the content of the paper.

## Figures and Tables

**Table 1:** Key behavioural categories, specific behaviours and quantifiable effects associated with health and welfare challenges that may have potential for automated detection.

Behavioural category	Specific behaviour(s)	Quantifiable effect(s)
Daily activity budget	Group of behaviours in a budget	Change in pattern (Salak-Johnson et al, 2004, Escobar et al, 2007, Reiner et al, 2009, Statham et al, 2009, Maselyne et al, 2014a); Interaction with enrichment (Trickett et al., 2009); Change in behavioural complexity (Rutherford et al., 2006).
Feeding, drinking, and elimination	Feed intake	Reduction in intake (Jackson, Cockcroft, 2007b, Kyriazakis, Houdijk, 2007, Kyriazakis, 2014, Ahmed et al, 2015).
	Change in pattern of intake	Change in frequency and/or duration of eating/drinking (Tolkamp et al, 2011, Andersen et al, 2014).
	Water intake	Reduction in intake (Madsen, Kristensen, 2005, Averos, 2007, Seddon, 2011, Rushen et al, 2012).
	Defecation	Constipation or diarrhoea, straining, cleanliness (Krsnik et al, 1999, Madsen, Kristensen, 2005, Radostits et al, 2007f, Rostagno et al, 2011, Ahmed et al, 2015).
	Urinary frequency, diuretic diuresis, stasis	Change in frequency/volume (Radostits et al., 2007b).
Posture and locomotion	Walking	Lameness scoring (D'Eath, 2012); Change in gait (Taylor, 1999); Circling or walking into objects (Radostits et al., 2007e).
	Sitting	Guarding and dog sitting (Radostits et al., 2007d).
	Lying	Duration lying (Rostagno et al., 2011).
	Tail position	Tails up (Kleinbeck and McGlone, 1993); Tails pressed between hind legs (Kiley-Worthington, 1976, Noonan et al, 1994).
Social behaviour	Cohesion or isolation	Deliberate clustering (Jackson, Cockcroft, 2007a, Cook et al, 2015); or separation from others (Reimert et al., 2013).
	Vocalisation	Frequency, duration, or amplitude call rate (Manteuffel et al, 2004, Moura et al, 2008, Vandermeulen et al, 2015).
	Tail biting	Change in activity levels pre outbreak (Statham et al., 2009); Increased chewing behaviour (Ursinus et al., 2014); Tail held in tucked position (Wallenbeck and Keeling, 2013).
Disease-specific behaviours	Coughing	Presence in respiratory infection (Ferrari et al., 2008).
	Scratching	Pruritic mange (Taylor, 1999).

**Table 2:** Current levels of automation for each behavioural category.

Behavioural categories	Automation categories		
	Behaviour detection	Behaviour monitoring	Automated detection of behavioural change
Daily activity budget	Requires monitoring behaviour over time	Location-based (Andersen et al., 2014); Locomotor activity (Chung et al., 2014); Drinking (Madsen and Kristensen, 2005).	
Feeding, drinking, and elimination	Drinking (Meiszberg et al., 2009).	Feeding (Fernández et al, 2011, Andersen et al, 2014, Maselyne et al, 2015b); Commercial systems: I-BOX 360°, Farmex, Eliskool 2/Elistar 2/Tristar systems, IVOG, FIRE; Elimination (Zhu et al., 2009).	Drinking (Madsen and Kristensen, 2005).
Posture and locomotion	Locomotion (Lind et al, 2005, Kongsro, 2013).	Spatial distribution (Cook et al, 2015, Nasirahmadi et al, 2015); Gait (Stavrakakis et al., 2014); General activity (Leroy et al., 2006).	Activity (Martínez-Avilés et al., 2015).
Social behaviour	Aggression (Oczak et al, 2012, Oczak et al, 2014, Viazzi et al, 2014, Lee et al, 2016); Clustering (Shao and Xin, 2008); Vocalisation (Manteuffel, Schön, 2002, Schön et al, 2004).		
Disease-specific	Coughing (Chedad et al, 2001, Exadaktylos et al, 2008, Chung et al, 2013).	Coughing (Vandermeulen et al, 2013, Hemeryck, Berckmans, 2015, Hemeryck et al, 2015).	

## Footnotes

1 See: Farm Animal Welfare Council press statement, December 5 1979. http://webarchive.nationalarchives.gov.uk/20121007104210/http://www.fawc.org.uk/pdf/fivefreedoms1979.pdf (accessed 20 September 2016).

2 See: Vicon Motion Systems, 2016. https://www.vicon.com/ (accessed 20 September 2016).

3 See: Skiold Acemo, 2016. Breeding Equipment. ESF station for sows and fattening pigs. http://www.skiold-acemo.com/en/breeding-equipment-uk (accessed 20 September 2016).

4 See: Hokofarm Group, 2016. http://www.hokofarmgroup.com/company/history.aspx (accessed 20 September 2016).

5 See: Insentec, 2013. Focus on Efficiency. http://archive-eu.com/page/2991500/2013-10-09/http://www.insentec.eu/en/pig-mgt/ivog (accessed 20 September 2016).

6 See: Osborne Industries, Inc., 2016. FIRE Pig Feeder. https://www.osbornelivestockequipment.com/products/fire-pig-performance-testing-system/fire-pig-feeder/ (accessed 20 September 2016).

7 See Footnote 3.

8 See: Automated Production Systems, 2016. I-BOX 360° Information System. http://www.automatedproduction.com/en/apibox.php (accessed 20 September 2016).

9 See: Farmex, 2016. Farm Energy and Control Services Ltd. http://www.farmex.co.uk/ (accessed 20 September 2016).

10 See: FANCOM, 2016. EyeNamic behaviour monitor http://www.fancom.com/en/broilers/biometrics (accessed 20 September 2016).

11 See: SoundTalks NV, 2016. Pig respiratory distress package. Respiratory distress monitor (RDM). http://www.soundtalks.be (accessed 20 September 2016).

## References

[bib0010] Aarnink A.J.A., Schrama J.W., Heetkamp M.J.W., Stefanowska J., Huynh T.T.T. (2006). Temperature and body weight affect fouling of pig pens. Journal of Animal Science.

[bib0015] Ahmed S.T., Mun H.-S., Yoe H., Yang C.-J. (2015). Monitoring of behavior using a video-recording system for recognition of Salmonella infection in experimentally infected growing pigs. Animal : An International Journal of Animal Bioscience.

[bib0020] Ahrendt P., Gregersen T., Karstoft H. (2011). Development of a real-time computer vision system for tracking loose-housed pigs. Computers and Electronics in Agriculture.

[bib0025] Andersen H.M.-L., Dybkjær L., Herskin M.S. (2014). Growing pigs' drinking behaviour: Number of visits, duration, water intake and diurnal variation. Animal: An International Journal of Animal Bioscience.

[bib0030] Averos X. (2007). Serum stress parameters in pigs transported to slaughter under commercial conditions in different seasons. Veterinarni Medicina.

[bib0035] Banhazi T., Vranken E., Berckmans D., Rooijakkers L., Berckmans D. (2015). Word of caution for technology providers: Practical problems associated with large scale deployment of PLF technologies on commercial farms. Precision Livestock Farming Applications.

[bib0040] Brouček J. (2014). Effect of noise on performance, stress, and behaviour of animals. Slovak Journal of Animal Science.

[bib0045] Brown-Brandl T.M., Eigenberg R.A. (2011). Development of a livestock feeding behavior monitoring system. Transactions of the ASABE.

[bib0050] Bruininx E.M.A.M., Van Der Peet-Schwering C.M.C., Schrama J.W., Den Hartog L.A., Everts H., Beynen A.C. (2001). The IVOG® feeding station: A tool for monitoring the individual feed intake of group-housed weanling pigs. Journal of Animal Physiology and Animal Nutrition.

[bib0055] Chapinal N., de Passillé A.M., Rushen J. (2010). Correlated changes in behavioral indicators of lameness in dairy cows following hoof trimming. Journal of Dairy Science.

[bib0060] Chedad A., Moshou D., Aerts J.M., Van Hirtum A., Ramon H., Berckmans D. (2001). Recognition system for pig cough based on probabilistic neural networks. Journal of Agricultural Engineering Research.

[bib0065] Chung Y., Oh S., Lee J., Park D., Chang H.-H., Kim S. (2013). Automatic detection and recognition of pig wasting diseases using sound data in audio surveillance systems. Sensors.

[bib0070] Chung Y., Kim H., Lee H., Park D., Jeon T., Chang H.-H. (2014). A cost-effective pigsty monitoring system based on a video sensor. KSII Transactions on Internet and Information Systems.

[bib0075] Cook N.J., Chabot B., Lui T., Bench C.J., Schaefer A.L. (2015). Infrared thermography detects febrile and behavioural responses to vaccination of weaned piglets. Animal : An International Journal of Animal Bioscience.

[bib0080] Cornou C., Kristensen A.R. (2013). Use of information from monitoring and decision support systems in pig production: Collection, applications and expected benefits. Livestock Science.

[bib0085] Costa A., Mentasti T., Guarino M., Leroy T., Berckmans D. (2007). Real time monitoring of pig activity: practical difficulties in pigs' behaviour labelling. Precision Livestock Farming '07.

[bib0090] Costa A., Borgonovo F., Leroy T., Berckmans D., Guarino M. (2009). Dust concentration variation in relation to animal activity in a pig barn. Biosystems Engineering.

[bib0095] Costa A., Ismayilova G., Borgonovo F., Viazzi S., Berckmans D., Guarino M. (2014). Image-processing technique to measure pig activity in response to climatic variation in a pig barn. Animal Production Science.

[bib0100] Dantzer R. (2004). Cytokine-induced sickness behaviour: A neuroimmune response to activation of innate immunity. European Journal of Pharmacology.

[bib0105] Davies E.R. (2009). The application of machine vision to food and agriculture: A review. The Imaging Science Journal.

[bib0110] D'Eath R. (2012). Repeated locomotion scoring of a sow herd to measure lameness: Consistency over time, the effect of sow characteristics and inter-observer reliability. Animal Welfare.

[bib0115] Edwards S.A. (2006). Tail biting in pigs: Understanding the intractable problem. The Veterinary Journal.

[bib0120] Eigenberg R.A., Brown-Brandl T.M., Nienaber J.A. (2008). Sensors for dynamic physiological measurements. Computers and Electronics in Agriculture.

[bib0125] Escobar J., Van Alstine W.G., Baker D.H., Johnson R.W. (2007). Behaviour of pigs with viral and bacterial pneumonia. Applied Animal Behaviour Science.

[bib0130] Exadaktylos V., Silva M., Aerts J.-M., Taylor C.J., Berckmans D. (2008). Real-time recognition of sick pig cough sounds. Computers and Electronics in Agriculture.

[bib0135] Feddes J.J.R., Fraser D., Buckley D.J., Poirier P. (1993). Electronic sensing of non-destructive chewing by growing pigs. Transactions of the ASAE.

[bib0140] Fernández J., Fàbrega E., Soler J., Tibau J., Ruiz J.L., Puigvert X., Manteca X. (2011). Feeding strategy in group-housed growing pigs of four different breeds. Applied Animal Behaviour Science.

[bib0145] Ferrari S., Silva M., Guarino M., Aerts J.M., Berckmans D. (2008). Cough sound analysis to identify respiratory infection in pigs. Computers and Electronics in Agriculture.

[bib0150] Figueroa J., Solà-Oriol D., Manteca X., Pérez J.F. (2013). Social learning of feeding behaviour in pigs: Effects of neophobia and familiarity with the demonstrator conspecific. Applied Animal Behaviour Science.

[bib0155] Frost A.R., Schofield C.P., Beaulah S.A., Mottram T.T., Lines J.A., Wathes C.M. (1997). A review of livestock monitoring and the need for integrated systems. Computers and Electronics in Agriculture.

[bib0160] González L.A., Tolkamp B.J., Coffey M.P., Ferret A., Kyriazakis I. (2008). Changes in feeding behavior as possible indicators for the automatic monitoring of health disorders in dairy cows. Journal of Dairy Science.

[bib0165] Gregersen T., Jensen T., Andersen M., Mortensen L., Maselyne J., Hessel E., Ahrendt P. (2013). Consumer grade range cameras for monitoring pig feeding behaviour.

[bib0170] Hart B.L. (1990). Behavioral adaptations to pathogens and parasites: Five strategies. Neuroscience & Biobehavioral Reviews.

[bib0175] Hemeryck M., Berckmans D. (2015). Pig cough monitoring in the EU-PLF project: first results. Precision Livestock Farming Applications.

[bib0180] Hemeryck M., Berckmans D., Vranken E., Tullo E., Fontana I., Guarino M., van Waterschoot T. (2015). The Pig Cough Monitor in the EU-PLF project results and multimodal data analysis in two case studies.

[bib0185] Hemsworth P.H., Coleman G.J., Barnett J.L., Borg S. (2000). Relationships between human-animal interactions and productivity of commercial dairy cows. Journal of Animal Science.

[bib0190] Hennecke M., Plotz T., Fink G.A., Schmalenstroer J., Hab-Umbach R. (2009). A hierarchical approach to unsupervised shape calibration of microphone array networks.

[bib0195] Hulsen J., Scheepens K. (2006). Pig Signals: Look, Think and Act.

[bib0200] Ismayilova G., Costa A., Fontana I., Berckmans D., Guarino M. (2013). Labelling the behaviour of piglets and activity monitoring from video as a tool of assessing interest in different environmental enrichments. Annals of Animal Science.

[bib0205] Jackson P.G., Cockcroft P.D. (2007). Handbook of Pig Medicine.

[bib0210] Jackson P.G., Cockcroft P.D. (2007). Handbook of Pig Medicine.

[bib0215] Jackson P.G., Cockcroft P.D. (2007). Handbook of Pig Medicine.

[bib0220] Jackson P.G., Cockcroft P.D. (2007). Handbook of Pig Medicine.

[bib0225] Jensen M.B., Kyriazakis I., Lawrence A.B. (1993). The activity and straw directed behaviour of pigs offered foods with different crude protein content. Applied Animal Behaviour Science.

[bib0230] Kashiha M., Bahr C., Haredasht S.A., Ott S., Moons C.P.H., Niewold T.A., Ödberg F.O., Berckmans D. (2013). The automatic monitoring of pigs water use by cameras. Computers and Electronics in Agriculture.

[bib0235] Kashiha M., Bahr C., Ott S., Moons C.P.H., Niewold T.A., Ödberg F.O., Berckmans D. (2013). Automatic identification of marked pigs in a pen using image pattern recognition. Computers and Electronics in Agriculture.

[bib0240] Kashiha M., Bahr C., Ott S., Moons C.P.H., Niewold T.A., Tuyttens F., Berckmans D. (2013). Automatic monitoring of pig activity using image analysis.

[bib0245] Kiley-Worthington M. (1976). The tail movements of ungulates, canids and felids with particular reference to their causation and function as displays. Behaviour.

[bib0250] Kleinbeck S., McGlone J.J. (1993). Pig tail posture: A measure of stress.

[bib0255] Kongsro J. (2013). Development of a computer vision system to monitor pig locomotion. Open Journal of Animal Sciences.

[bib0260] Krsnik B., Yammine R., Pavičić Ž., Balenović T., Njari B., Vrbanac I., Valpotić I. (1999). Experimental model of enterotoxigenic Escherichia coli infection in pigs: Potential for an early recognition of colibacillosis by monitoring of behavior. Comparative Immunology, Microbiology and Infectious Diseases.

[bib0265] Kulikov V.A., Khotskin N.V., Nikitin S.V., Lankin V.S., Kulikov A.V., Trapezov O.V. (2014). Application of 3-D imaging sensor for tracking minipigs in the open field test. Journal of Neuroscience Methods.

[bib0270] Kyriazakis I. (2014). Pathogen-induced anorexia: A herbivore strategy or an unavoidable consequence of infection?. Animal Production Science.

[bib0275] Kyriazakis I., Houdijk J. (2007). Food intake and performance of pigs during health, disease and recovery.

[bib0280] Kyriazakis I., Tolkamp B.J. (2010). Disease. The Encyclopedia of Applied Animal Behaviour and Welfare.

[bib0285] Kyriazakis I., Tolkamp B.J., Hutchings M.R. (1998). Towards a functional explanation for the occurrence of anorexia during parasitic infections. Animal Behaviour.

[bib0290] Larsen M.L.V., Andersen H.M.-L., Pedersen L.J. (2016). Can tail damage outbreaks in the pig be predicted by behavioural change?. The Veterinary Journal.

[bib0295] Lee J., Jin L., Park D., Chung Y. (2016). Automatic recognition of aggressive behavior in pigs using a kinect depth sensor. Sensors.

[bib0300] Leroy T., Mentasti T., Costa A., Guarino M., Aerts J.-M., Berckmans D. (2006). Eyenamic: Real-time measurement of pig activity in practical conditions.

[bib0305] Lind N.M., Vinther M., Hemmingsen R.P., Hansen A.K. (2005). Validation of a digital video tracking system for recording pig locomotor behaviour. Journal of Neuroscience Methods.

[bib0310] Madsen T.N., Kristensen A.R. (2005). A model for monitoring the condition of young pigs by their drinking behaviour. Computers and Electronics in Agriculture.

[bib0315] Manteuffel G., Schön P.C. (2002). Measuring pig welfare by automatic monitoring of stress calls. Bornimer Agrartechnische Berichte.

[bib0320] Manteuffel G., Puppe B., Schön P.C. (2004). Vocalization of farm animals as a measure of welfare. Applied Animal Behaviour Science.

[bib0325] Martínez-Avilés M., Fernández-Carrión E., López García-Baones J.M., Sánchez-Vizcaíno J.M. (2015). Early detection of infection in pigs through an online monitoring system. Transboundary and Emerging Diseases.

[bib0330] Marx G., Horn T., Thielebein J., Knubel B., von Borell E. (2003). Analysis of pain-related vocalization in young pigs. Journal of Sound and Vibration.

[bib0335] Maselyne J., Saeys W., De Ketelaere B., Briene P., Millet S., Tuyttens F., Van Nuffel A. (2014). How do fattening pigs spend their day?.

[bib0340] Maselyne J., Saeys W., De Ketelaere B., Mertens K., Vangeyte J., Hessel E.F., Millet S., Van Nuffel A. (2014). Validation of a high frequency radio frequency identification (HF RFID) system for registering feeding patterns of growing-finishing pigs. Computers and Electronics in Agriculture.

[bib0345] Maselyne J., Van Nuffel A., De Ketelaere B., Vangeyte J., Hessel E.F., Sonck B., Saeys W. (2014). Range measurements of a high frequency radio frequency identification (HF RFID) system for registering feeding patterns of growing–finishing pigs. Computers and Electronics in Agriculture.

[bib0350] Maselyne J., Adriaens I., Huybrechts T., Ketelaere B., de Millet S., Vangeyte J., Nuffel A., van Saeys W. (2015). Assessing the drinking behaviour of individual pigs using RFID registrations. Precision Livestock Farming Applications.

[bib0355] Maselyne J., Saeys W., Van Nuffel A. (2015). Review: Quantifying animal feeding behaviour with a focus on pigs. Physiology & Behavior.

[bib0360] McFarlane N.J.B., Schofield C.P. (1995). Segmentation and tracking of piglets in images. Machine Vision and Applications.

[bib0365] McManus C., Tanure C.B., Peripolli V., Seixas L., Fischer V., Gabbi A.M., Menegassi S.R.O., Stumpf M.T., Kolling G.J., Dias E. (2016). Infrared thermography in animal production: An overview. Computers and Electronics in Agriculture.

[bib0370] Meijer E., Bertholle C.P., Oosterlinck M., van der Staay F., Back W., van Nes A. (2014). Pressure mat analysis of the longitudinal development of pig locomotion in growing pigs after weaning. BMC Veterinary Research.

[bib0375] Meiszberg A.M., Johnson A.K., Sadler L.J., Carroll J.A., Dailey J.W., Krebs N. (2009). Drinking behavior in nursery pigs: Determining the accuracy between an automatic water meter versus human observers. Journal of Animal Science.

[bib0380] Mellor D. (2016). Updating animal welfare thinking: Moving beyond the ‘Five Freedoms’ towards ‘A Life Worth Living. Animals.

[bib0385] Moura D.J., Silva W.T., Naas I.A., Tolón Y.A., Lima K.A.O., Vale M.M. (2008). Real time computer stress monitoring of piglets using vocalization analysis. Computers and Electronics in Agriculture.

[bib0390] Munsterhjelm C., Heinonen M., Valros A. (2015). Effects of clinical lameness and tail biting lesions on voluntary feed intake in growing pigs. Livestock Science.

[bib0395] Nasirahmadi A., Richter U., Hensel O., Edwards S., Sturm B. (2015). Using machine vision for investigation of changes in pig group lying patterns. Computers and Electronics in Agriculture.

[bib0400] Nathues H., Spergser J., Rosengarten R., Kreienbrock L., Grosse Beilage E. (2012). Value of the clinical examination in diagnosing enzootic pneumonia in fattening pigs. The Veterinary Journal.

[bib0405] National Research Council (1981). Effect of Environment on Nutrient Requirements of Domestic Animals.

[bib0410] Nilsson M., Herlin A.H., Ardö H., Guzhva O., Åström K., Bergsten C. (2015). Development of automatic surveillance of animal behaviour and welfare using image analysis and machine learned segmentation technique. Animal: An International Journal of Animal Bioscience.

[bib0415] Nof S.Y. (2009). Automation: What it means to us around the world. Springer Handbook of Automation.

[bib0420] Noonan G.J., Rand J.S., Priest J., Ainscow J., Blackshaw J.K. (1994). Behavioural observations of piglets undergoing tail docking, teeth clipping and ear notching. Applied Animal Behaviour Science.

[bib0425] Oczak M., Costa A.M., Ismailova G., Sonoda L.T., Fels M., Hartung J., Guarino M., Viazzi S. (2012). Analysis of sequences in aggressive interactions of pigs for the development of an automatic aggression monitoring and control system.

[bib0430] Oczak M., Viazzi S., Ismayilova G., Sonoda L.T., Roulston N., Fels M., Bahr C., Hartung J., Guarino M., Berckmans D. (2014). Classification of aggressive behaviour in pigs by activity index and multilayer feed forward neural network. Biosystems Engineering.

[bib0435] Radostits O.M., Gay C.C., Hinchcliff K.W., Constable P.D. (2007). Veterinary Medicine.

[bib0440] Radostits O.M., Gay C.C., Hinchcliff K.W., Constable P.D. (2007). Veterinary Medicine.

[bib0445] Radostits O.M., Gay C.C., Hinchcliff K.W., Constable P.D. (2007). Veterinary Medicine.

[bib0450] Radostits O.M., Gay C.C., Hinchcliff K.W., Constable P.D. (2007). Veterinary Medicine.

[bib0455] Radostits O.M., Gay C.C., Hinchcliff K.W., Constable P.D. (2007). Veterinary Medicine.

[bib0460] Radostits O.M., Gay C.C., Hinchcliff K.W., Constable P.D. (2007). Veterinary Medicine.

[bib0465] Reimert I., Bolhuis J.E., Kemp B., Rodenburg T.B. (2013). Indicators of positive and negative emotions and emotional contagion in pigs. Physiology & Behavior.

[bib0470] Reiner G., Hübner K., Hepp S. (2009). Suffering in diseased pigs as expressed by behavioural, clinical and clinical-chemical traits, in a well defined parasite model. Applied Animal Behaviour Science.

[bib0475] Rostagno M.H., Eicher S.D., Lay D.C. (2011). Immunological, physiological, and behavioral effects of Salmonella enterica carriage and shedding in experimentally infected finishing pigs. Foodborne Pathogens and Disease.

[bib0480] Rushen J., Chapinal N., de Passillé A. (2012). Automated monitoring of behavioural-based animal welfare indicators. Animal Welfare.

[bib0485] Rutherford K.M.D., Haskell M.J., Glasbey C., Lawrence A.B. (2006). The responses of growing pigs to a chronic-intermittent stress treatment. Physiology & Behavior.

[bib0490] Sa J., Han S., Lee S., Kim H., Lee S., Chung Y., Park D. (2015). Image segmentation of adjoining pigs using spatio-temporal information. KIPS Transactions on Software and Data Engineering.

[bib0495] Salak-Johnson J.L., Anderson D.L., McGlone J.J. (2004). Differential dose effects of central CRF and effects of CRF astressin on pig behavior. Physiology & Behavior.

[bib0500] Sandberg F.B., Emmans G.C., Kyriazakis I. (2007). Partitioning of limiting protein and energy in the growing pig: Description of the problem, possible rules and their qualitative evaluation. British Journal of Nutrition.

[bib0505] Schön P.C., Puppe B., Manteuffel G. (2004). Automated recording of stress vocalisations as a tool to document impaired welfare in pigs. Animal Welfare.

[bib0510] Schrøder-Petersen D.L., Simonsen H.B. (2001). Tail biting in pigs. The Veterinary Journal.

[bib0515] Seddon Y.M. (2011). Development of improved disease monitoring tools and management strategies to promote health in finishing pigs.

[bib0520] Shao B., Xin H. (2008). A real-time computer vision assessment and control of thermal comfort for group-housed pigs. Computers and Electronics in Agriculture.

[bib0525] Silva M., Ferrari S., Costa A., Aerts J.-M., Guarino M., Berckmans D. (2008). Cough localization for the detection of respiratory diseases in pig houses. Computers and Electronics in Agriculture.

[bib0530] Soerensen D., Pedersen L. (2015). Infrared skin temperature measurements for monitoring health in pigs: A review. Acta Veterinaria Scandinavica.

[bib0535] Sonoda L.T., Fels M., Oczak M., Vranken E., Ismayilova G., Guarino M., Viazzi S., Bahr C., Berckmans D., Hartung J. (2013). Tail biting in pigs – Causes and management intervention strategies to reduce the behavioural disorder. A review. Berliner und Münchener tierärztliche Wochenschrift.

[bib0540] Statham P., Green L., Bichard M., Mendl M. (2009). Predicting tail-biting from behaviour of pigs prior to outbreaks. Applied Animal Behaviour Science.

[bib0545] Stavrakakis S., Guy J.H., Warlow O.M.E., Johnson G.R., Edwards S.A. (2014). Walking kinematics of growing pigs associated with differences in musculoskeletal conformation, subjective gait score and osteochondrosis. Livestock Science.

[bib0550] Stavrakakis S., Li W., Guy J.H., Morgan G., Ushaw G., Johnson G.R., Edwards S.A. (2015). Validity of the Microsoft Kinect sensor for assessment of normal walking patterns in pigs. Computers and Electronics in Agriculture.

[bib0555] Studnitz M., Jensen M.B., Pedersen L.J. (2007). Why do pigs root and in what will they root?. Applied Animal Behaviour Science.

[bib0560] Szeliski R. (2011). Computer Vision.

[bib0565] Tarrés J., Tibau J., Piedrafita J., Fàbrega E., Reixach J. (2006). Factors affecting longevity in maternal Duroc swine lines. Livestock Science.

[bib0570] Taylor D.J. (1999). Pig Diseases.

[bib0575] Taylor N.R., Main D.C.J., Mendl M., Edwards S.A. (2010). Tail-biting: A new perspective. The Veterinary Journal.

[bib0580] Tolkamp B.J., Allcroft D.J., Barrio J.P., Bley T.A.G., Howie J.A., Jacobsen T.B., Morgan C.A., Schweitzer D.P.N., Wilkinson S., Yeates M.P. (2011). The temporal structure of feeding behavior. AJP: Regulatory, Integrative and Comparative Physiology.

[bib0585] Trickett S.L., Guy J.H., Edwards S.A. (2009). The role of novelty in environmental enrichment for the weaned pig. Applied Animal Behaviour Science.

[bib0590] Ursinus W.W., Van Reenen C.G., Kemp B., Bolhuis J.E. (2014). Tail biting behaviour and tail damage in pigs and the relationship with general behaviour: Predicting the inevitable?. Applied Animal Behaviour Science.

[bib0595] Uzal S., Ugurlu N. (2010). The dairy cattle behaviors and time budget and barn area usage in freestall housing. Journal of Animal and Veterinary Advances.

[bib0600] Vandermeulen J., Decré W., Berckmans D., Exadaktylos V., Bahr C., Berckmans D. (2013). The pig cough monitor: from research topic to commercial product.

[bib0605] Vandermeulen J., Bahr C., Tullo E., Fontana I., Ott S., Kashiha M., Guarino M., Moons C.P.H., Tuyttens F.A.M., Niewold T.A. (2015). Discerning pig screams in production environments. PLoS ONE.

[bib0610] Viazzi S., Ismayilova G., Oczak M., Sonoda L.T.T., Fels M., Guarino M., Vranken E., Hartung J., Bahr C., Berckmans D. (2014). Image feature extraction for classification of aggressive interactions among pigs. Computers and Electronics in Agriculture.

[bib0615] Wallenbeck A., Keeling L.J. (2013). Using data from electronic feeders on visit frequency and feed consumption to indicate tail biting outbreaks in commercial pig production. Journal of Animal Science.

[bib0620] Wathes C.M., Kristensen H.H., Aerts J.-M., Berckmans D. (2008). Is precision livestock farming an engineer's daydream or nightmare, an animal's friend or foe, and a farmer's panacea or pitfall?. Computers and Electronics in Agriculture.

[bib0625] Zebunke M., Puppe B., Langbein J. (2013). Effects of cognitive enrichment on behavioural and physiological reactions of pigs. Physiology & Behavior.

[bib0630] Zhu W., Pu X., Li X., Zhu X. (2009). Automated detection of sick pigs based on machine vision.

[bib0635] Zonderland J.J., Vermeer H.M., Vereijken P.F.G., Spoolder H.A.M. (2003). Measuring a pig's preference for suspended toys by using an automated recording technique. Agricultural Engineering International: CIGR Journal.

